# Structural and Functional Characterization of Medicinal Plants as Selective Antibodies towards Therapy of COVID-19 Symptoms

**DOI:** 10.3390/antib13020038

**Published:** 2024-05-07

**Authors:** Fatemeh Mollaamin

**Affiliations:** Department of Biomedical Engineering, Faculty of Engineering and Architecture, Kastamonu University, Kastamonu 37150, Turkey; fmollaamin@kastamonu.edu.tr or smollaamin@gmail.com

**Keywords:** natural medication, COVID-19 treatment, achillin, alkannin, cuminaldehyde, dillapiole, estragole, fenchone, medicinal plant, DFT

## Abstract

Considering the COVID-19 pandemic, this research aims to investigate some herbs as probable therapies for this disease. *Achillea millefolium* (*Yarrow*), *Alkanet*, *Rumex patientia* (*Patience dock*), *Dill*, *Tarragon*, and *sweet fennel*, including some principal chemical compounds of achillin, alkannin, cuminaldehyde, dillapiole, estragole, and fenchone have been selected. The possible roles of these medicinal plants in COVID-19 treatment have been investigated through quantum sensing methods. The formation of hydrogen bonding between the principal substances selected in anti-COVID natural drugs and Tyr-Met-His (the database amino acids fragment), as the active area of the COVID protein, has been evaluated. The physical and chemical attributes of nuclear magnetic resonance, vibrational frequency, the highest occupied molecular orbital energy and the lowest unoccupied molecular orbital energy, partial charges, and spin density have been investigated using the DFT/TD-DFT method and 6-311+G (2d,p) basis set by the Gaussian 16 revision C.01 program toward the industry of drug design. This research has exhibited that there is relative agreement among the results that these medicinal plants could be efficient against COVID-19 symptoms.

## 1. Introduction

The COVID-19 pandemic is a serious malady caused by a new coronavirus known as severe acute respiratory syndrome (SARS-CoV-2). There are no trustworthy remedies or acceptable vaccines to fight against SARS-CoV-2. More attempts to probe for antiviral agents against COVID-19 are essential, while phytochemicals can be a powerful solution.

Natural medications are an important part of common health, notwithstanding the progress of the health system. In rural areas, local treatment keeps its importance as the primary procedure in the usual seasonal maladies, like colds and flu. The most important reason for using herbs and medicinal plant treatments is the belief that they will influence one’s health. Natural products from medicinal plants are, therefore, bringing hope in the form of phytocompounds, which can either kill SARS-CoV-2, interfere with its replication, or strengthen the human body’s immunity to fight against infection.

Recently, almost all antibodies have been produced in human cells and transformed animal cells. These are platforms that require a lot of equipment and take a long time to set up. Many plant-based antibodies can respond very quickly to the emergence of new variants of COVID-19. The emergence of a new coronavirus, known as SARS-CoV-2, has initiated a pandemic of COVID-19. Since its first reported case in Wuhan, China, in December 2019, new evidence discovered by both clinicians and researchers globally has helped to shed some light on the disease pathogenesis and the nature of the virus itself. The availability of new information has subsequently informed policy changes on transmission prevention strategies, as well as the development of preventative vaccines and therapeutic drug candidates. Enforced physical distancing, hand hygiene, and arguably, proper usage of personal protective equipment, including wearing a surgical mask, remains the most effective way of controlling the spread of the disease. Most countries that have adopted such measures have reported some success in curbing the spread of the disease [1,2,3,4,5,6,7,8,9,10].

In the research of phytomedicine, it is common to observe multiple pharmacological properties from a single plant. It is now well understood that a single plant may contain a wide range of phytochemicals, making ethnopharmacology research full of possibilities yet challenging. On top of exhibiting direct antiviral effects, medicinal plants with reported anti-inflammatory activities may have pleiotropic roles in COVID-19 management, such as the elevation of inflammatory markers [11,12,13,14,15,16,17,18,19].

*Achillea millefolium*, or *common yarrow*, is a flowering plant in the family *Asteraceae*. It is native to temperate regions of the Northern Hemisphere in Asia, Europe, and North America. It has been introduced as a feed for livestock in New Zealand and Australia, where it is a common weed growing in both wet and dry areas, such as roadsides, meadows, fields, and coastal locations. *Achillea millefolium* is used in traditional medicine, possibly due to its astringent effects. *Yarrow* and its North American varieties were traditionally used by many Native American nations. Native American nations used the plant for healing cuts and abrasions, for relief of earaches and throat infections, and as an eyewash. *Common yarrow* was used by plains Indigenous peoples to reduce pain or fever and aid sleep (Table 1) [20,21].

*Rumex patientia*, known as *patience dock*, [4] *garden patience*, *herb patience*, or *monk’s rhubarb*, is an herbaceous perennial flowering plant belonging to the family *Polygonaceae*. In spring, it is often consumed as a leaf vegetable and used as a filling in pies in Southern Europe, especially in Bulgaria, North Macedonia, and Serbia. It is also used in Romania in spring broths or sarmale. *Rumex patientia,* or *patience dock,* is an uncommon weed found growing on roadsides, farm fields, and waste areas. Some of the distinguishing characteristics of *Rumex patientia* are whether the leaves are crinkly wavy or relatively flat, the shape of the inner tepals at maturity, the size and shape of the grains, whether the grains on all three inner tepals are about the same size, sometimes the length of the flower stalk, whether the stalk is jointed, or whether a vein pattern appears on the leaves. *Patience dock* has weakly crinkly wavy leaves, tepals up to 8 mm long that range from kidney-shaped to nearly round and is slightly ragged around the edge, a single grain about a quarter as long as the tepal, and a flower stalk that has a swollen joint near the base. It has the largest tepals of the *Minnesota Rumex* species, and the (usually) single, small grain makes it unique (Table 1) [22].

*Dill* or *Anethum* graveolens is an annual herb in the celery family Apiaceae. It is the only species in the genus Anethum. *Dill* is grown widely in Eurasia, where its leaves and seeds are used as an herb or a spice for flavoring food. Dillapiole is a natural constituent of *Anethum graveolens*, which exhibits potential biological properties. Dillapiole may be used as an analytical reference standard for the quantification of the analyte in the French bean *Phaseolus* sp. Treated with pesticidal formulation, as well as in *dill*, *caraway seeds*, and pharmaceutical formulations using chromatography techniques (Table 1) [23,24].

One subspecies, *tarragon*, is cultivated for the use of its leaves as an aromatic culinary herb. From some other subspecies, a characteristic aroma is largely absent (Table 1) [25,26].

Sweet fennel is a flowering plant species in the carrot family. It is a hardy, perennial herb with yellow flowers and feathery leaves. It is indigenous to the shores of the Mediterranean but has become widely naturalized in many parts of the world, especially on dry soils near the seacoast and on riverbanks. It is a highly flavorful herb used in cooking and, along with the similar-tasting anise, is one of the primary ingredients of absinthe (Table 1) [27,28].

Flavonoids from natural medication were represented as possessing antiviral bioactivities [29,30,31]. In this work, it has been illustrated that achillin, alkannin, cuminaldehyde, dillapiole, estragole, and fenchone are the probable anti-COVID-19 receptor derived from medicinal plants (Table 1).

Based on this research, it can be estimated the occasions for discovering the efficient medication against COVID-19 using quantum mechanics computations to measure the effect of hydrogen bonding in the variety of junctions with these seven natural drugs of achillin, alkannin, cuminaldehyde, dillapiole, estragole and fenchone bound to the active area of COVID-19 virus [32,33,34,35,36,37].

## 2. Materials and Methods

Achillin, alkannin, cuminaldehyde, dillapiole, estragole, and fenchone have been attached to the active area of COVID-19 protein compounds, which approves the existence of hydrogen bonds toward resistant complexes. Therefore, quantum mechanics approaches with m062x/cc-pvdz pseudo=CEP function for complexes of seven inhibitors for COVID-19 have been accomplished. The favorable coordination of the optimized substances of phenilic natural drug joint to Tyr160-Met161-His162 with IR spectroscopy using the Gaussian 16 revision C.01 program package [38] has been measured due to the DFT method and m062x/cc-pvdz pseudo=CEP level of theory. The [Perdew–Burke–Ernzerhof] “PBE” functional with high-precision generalized gradient approximation “GGA” has been employed to achieve more authentic results [39].

It has been exhibited that polarization functions in the employed basis set in the calculation always reflect magnificent prosperity in simulation and modeling in the drug design industry [40,41,42,43,44,45,46,47]. Frequency achievement is the finding of harmonic potential wells by analytic procedures that keep the activity of all atoms at the same time in the vibration time scale, conducting an inherent illustration of vibrations in molecules [48,49,50,51,52].

Thus, the geometry optimization of coordination in medicinal extracts-TMH agents based on the drug design has been found from the active area of certain atoms of “O”, “N,” and “H” in the attachment of bond angle and torsion angle values (Table 2).

For carrying out a firm compound of natural medication attached to a COVID-19 active site, the chemical shift of nuclear magnetic resonance, vibrational frequency, and intensity of the normal modes have been computed with the “QM” methods, and the original vibrational modes have been analyzed [53,54,55,56,57,58].

Computational measurements have been carried out in a variety of theoretical levels to profit from the more precise balance of geometrical amounts and infrared spectral information for each of the indicated substances. It is assumed that further diffuse and polarization functions into the basis set employed in the calculation direct us to the high evolution of the results of methodical approaches [59,60,61,62].

The different approaches in modeling and simulation exhibit the path that can generate a usual model at a particular temperature by evaluating all physical and chemical attributes based on the partition function amounts [63,64,65,66,67,68,69,70,71].

## 3. Results and Discussion

### 3.1. Nuclear Magnetic Resonance (NMR) Analysis

**Figure 1:**
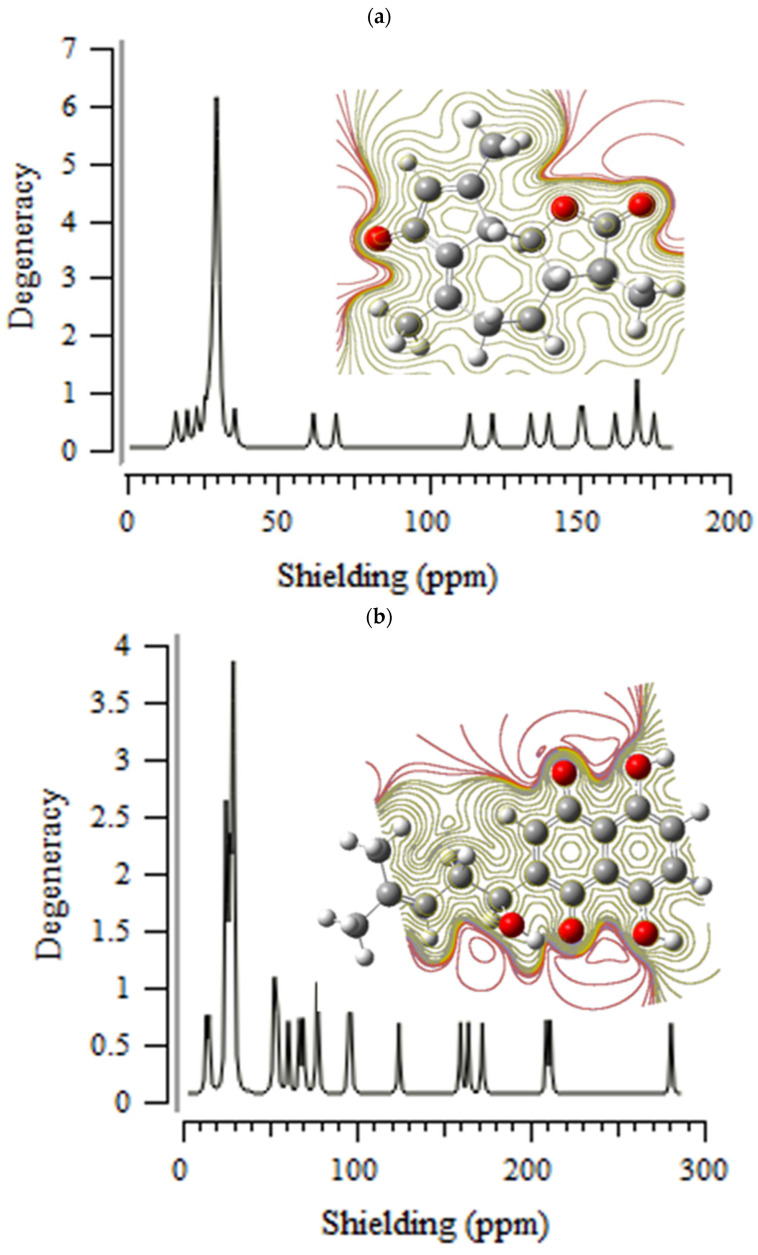

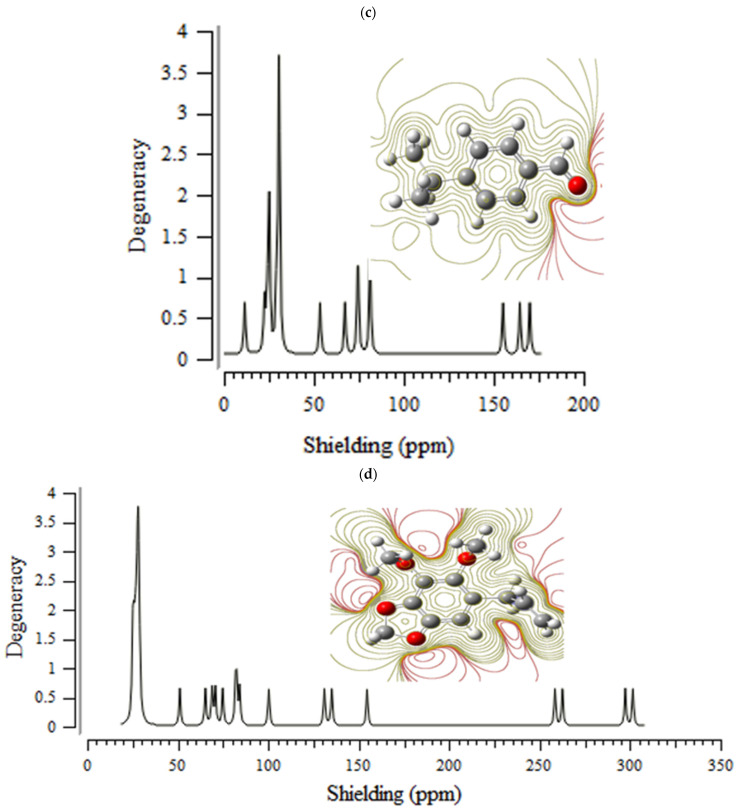

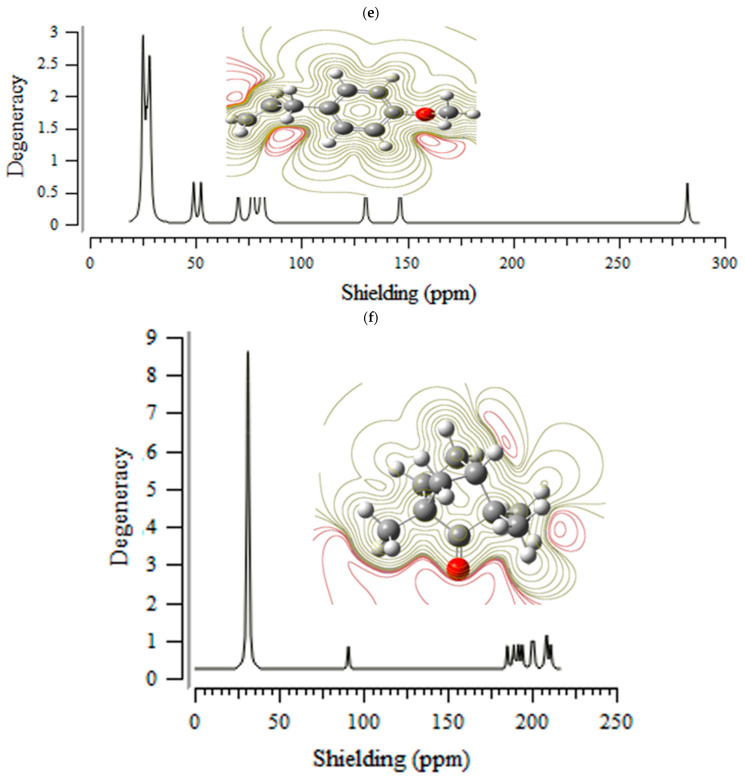
“NMR” spectroscopy for (**a**) achillin, (**b**) alkannin, (**c**) cuminaldehyde, (**d**) dillapiole, (**e**) estragole and (**f**) fenchone bound to “TMH” COVID-19 active area through the drug design approach.

The amounts of “NMR” shifts for Tyr160-Met161-His162 through the database of amino acids in beta-sheet conformation and four certain extracts of natural medications containing achillin, alkannin, cuminaldehyde, dillapiole, estragole, and fenchone have been evaluated to discover the exhibited of oxygen, nitrogen, and hydrogen in the active sites of these anti-virus medications through the production of hydrogen bonding by representing the reaction area of “TMH” agent (Figure 1a–f).

Achillin, alkannin, cuminaldehyde, dillapiole, estragole, and fenchone have approximately shown identical behavior (about 30–200 ppm) for different atoms in the interaction site of these substances with Tyr160-Met161-His162 through the “NMR” calculations accompanying electron spin density (ESP) (Figure 1). The strongest graph of “NMR” spectroscopy has been almost seen in 30 ppm for all principal ingredients of herbal medicine. The most fragile graphs of the “NMR” spectrum have approximately been observed between 50 and 200 ppm (Figure 1).

Nuclear magnetic resonance properties have denoted the critical points of essential extracts of pharmaceutical kinds for attaching to the Tyr160-Met161-His162 (TMH) in producing the anti-virus medications while each critical atom of “O” and “N” as the electronegative atoms for jointing to the hydrogen has remarked the major changing in the “NMR” graphs (Figure 1a–f).

### 3.2. Infrared (IR) Spectra Analysis and Thermodynamic Properties

**Figure 2:**
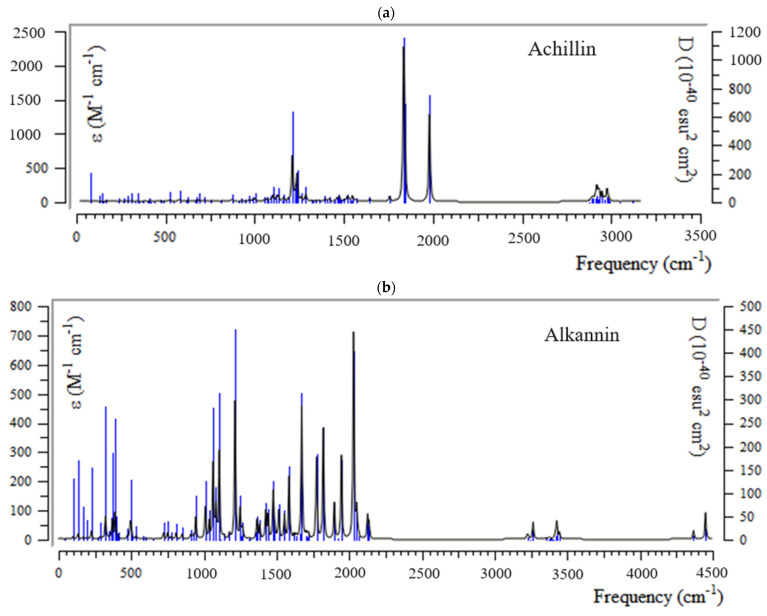

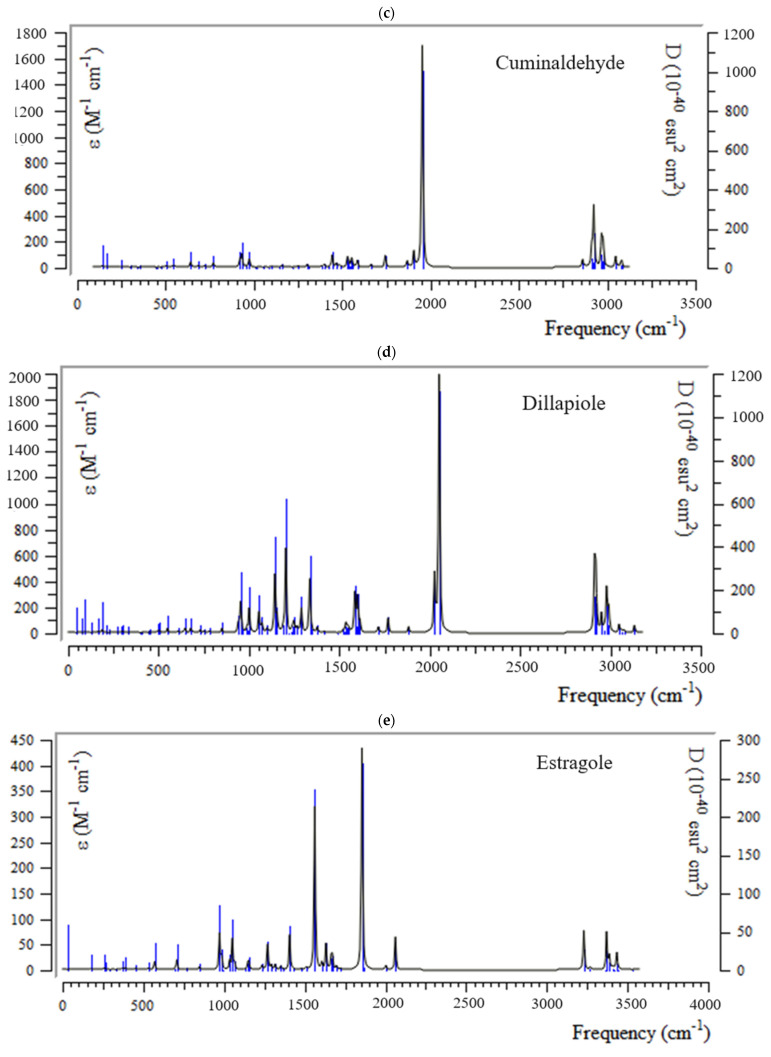

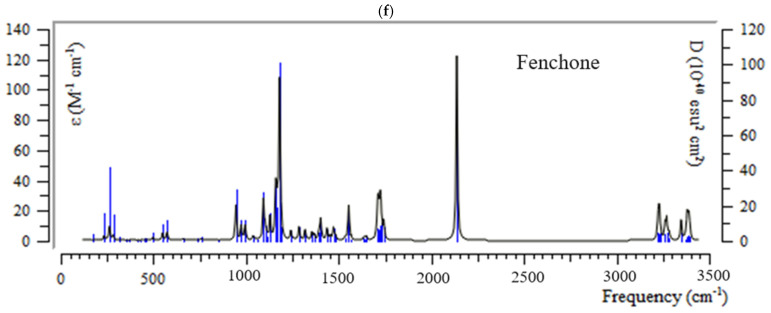
The graphs of “IR” spectra for (**a**) achillin, (**b**) alkannin, (**c**) cuminaldehyde, (**d**) dillapiole, (**e**) estragole, and (**f**) fenchone bound to “TMH” through the drug design approach achieved by m062x/cc-pvdz pseudo = CEP level of theory.

The IR calculations for main ingredients of medicinal plants including achillin, alkannin, cuminaldehyde, dillapiole, estragole and fenchone have been calculated for fixing the intersection of Tyr160-Met161-His162 as the anti-COVID-19 medication through the drug design approach applying “IR” spectroscopy using Gaussian 16 revision C.01 program to obtain the best amounts for geometrical coordination and thermochemical parameters (Figure 2a–f). The most fluctuation of frequency of “IR” spectra for alendronic acid, ibandronic acid, neridronic acid, and pamidronic acid has been approximately seen between 500 and 3000 cm^−1^. The strongest peaks of “IR” graph for principal ingredients of herbal medicine have been observed in 1850 cm^−1^ for achillin (Figure 2a), in 2000 cm^−1^ for alkannin (Figure 2b), in 1950 cm^−1^ for cuminaldehyde (Figure 2c), in 2050 cm^−1^ for dillapiole (Figure 2d), in 1850 cm^−1^ for estragole cm^−1^ (Figure 2e), and in 2125 cm^−1^ for fenchone (Figure 2f).

**Figure 3:**
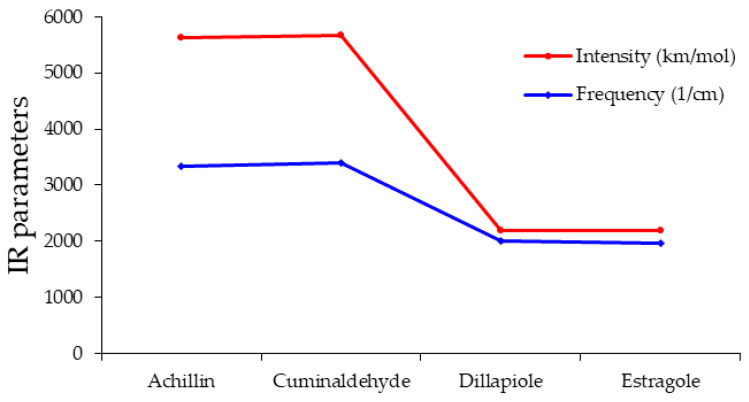
The curves of “IR” spectra for medicinal plants of achillin, cuminaldehyde, dillapiole, and estragole anti-COVID-19 drugs in normal mode = 59.

In achillin, cuminaldehyde, dillapiole, and estragole attached to Tyr160-Met161-His162 through its database of amino acids in beta-sheet conformation, as the critical point of COVID-19 protein compound in the procedure of drug design steps, the functions of frequency and intensity of diverse infrared normal modes of pharmaceutical extracts-TMH complexes have been discovered to be significantly distinct through the resistance of hydrogen bonding organized between the critical point of COVID-19 agent B.1.1.529 and pharmaceutical extracts which establishes the anti-COVID-19 medication (Table 3 and Figure 3).

The intensity and frequency of TMH attachment have been explored to vary dramatically with each pharmaceutical extract therapy consisting of achillin, cuminaldehyde, dillapiole, estragole, and fenchone. It has been seen that the frequency and intensity of achillin and cuminaldehyde are higher than dillapiole and estragole (Figure 3). Then, thermodynamic properties have distinguished the resistant anti-COVID-19 agent complexes of principal extracts of pharmaceutical kinds of “TMH” through the hydrogen bonding constitution employing the drug design framework (Table 4).

**Figure 4:**
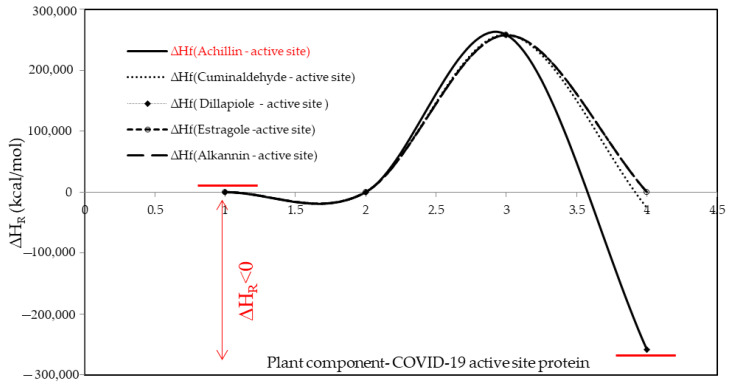
The changes in ∆H_R_ among achillin, alkannin, cuminaldehyde, dillapiole, and estragole bound to COVID-19 active site protein.

Moreover, the difference in ∆H_F_ among achillin, alkannin, cuminaldehyde, dillapiole, and estragole has been illustrated due to the formation of the hydrogen bonding in Tyr160-Met161-His162 agent of the critical point in COVID-19 macromolecule by analyzing the database of amino acids in beta-sheet conformation (Table 5 and Figure 4).

### 3.3. Charge Distribution

In this part, the atomic charge of certain atoms of “O” attachment of achillin, alkannin, cuminaldehyde, dillapiole, and estragole with Tyr160-Met161-His162 agent has been measured in the critical point of hydrogen bonding existence (Table 6).

**Figure 5:**
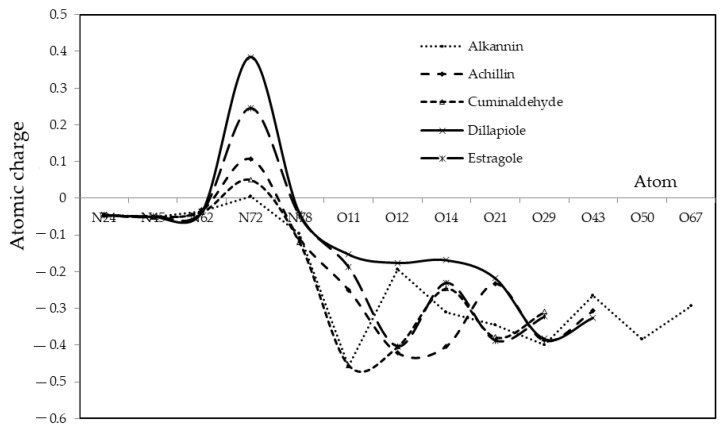
Comparing “Q” versus indicated “O” atoms in the attachment of active areas of achillin, alkannin, cuminaldehyde, dillapiole, and estragole with Tyr160-Met161-His162 agent.

Then, in Figure 5, it has been sketched the alterations of “Q” of indicated “O” atoms for optimized molecules of achillin, alkannin, cuminaldehyde, dillapiole, and estragole with Tyr160-Met161-His162 agent due to the existence of hydrogen bonding. Thus, the consequences of Table 6 in a polar area have notified the consistency of COVID-19 medications, which have been accomplished considering the oxygen as the electronegative atoms in the growth of the hydrogen bonding using the drug design insight, which has proposed the modeling of an anti-COVID-19 drug.

All in all, the prospect of Figure 5 suggests the proof for the existence of various “Q” on natural medication-COVID-19 complexes as the anti-COVID-19 drugs, which basically depends on the status of critical points of exhibited atoms of “O”, “N” and “H” in the attachment of bond angles. On the other hand, the spin density and partial charges have been achieved by matching the electrostatic potential to make up the charge of “O” and “N” with high electronegativity in the linkage of an electrophilic group of ‘‘H” in the compounds of natural medication-COVID-19 as the anti-virus drugs which can direct us toward the industry of drug design outlook.

### 3.4. HOMO and LUMO: Frontier Orbitals

“HOMO”, the highest occupied molecular orbital energy, and “LUMO”, the lowest unoccupied molecular orbital energy, have been calculated for some effective ingredients of achillin, alkannin, cuminaldehyde, dillapiole, estragole, and fenchone (Table 7). The HOMO, LUMO, and band energy gap (ev) indicated the pictorial explanation of the frontier molecular orbital and their respective positive and negative zones, which are an important factor for identifying the molecular characteristics of achillin, alkannin, cuminaldehyde, dillapiole, estragole and Fenchone in of six selected medicinal plants of *Achillea millefolium* (Yarrow), *Alkanet*, *Rumex patientia* (*Patience dock*), *Dill*, *Tarragon*, and *Sweet fennel*.

**Figure 6:**
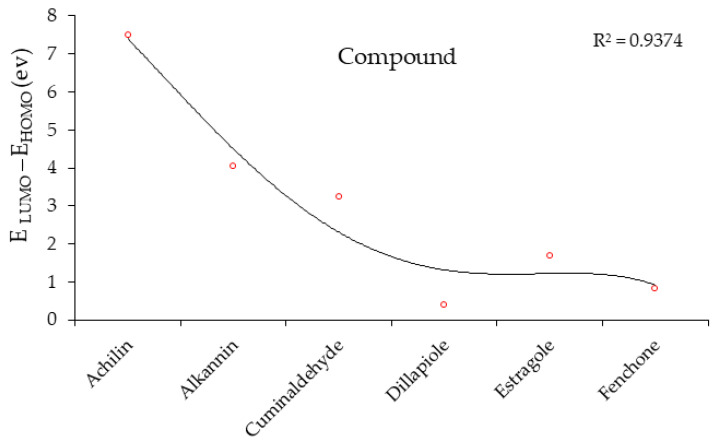
The band energy gap (eV) for achillin, alkannin, cuminaldehyde, dillapiole, estragole, and fenchone.

In fact, the “HOMO” presents the susceptibility for releasing an electron while the “LUMO” as an electron acceptor introduces the competence for receiving an electron particle. So, the energy gap (∆E = E LUMO–EHOMO) explains the energy diversity between the frontier “HOMO” and “LUMO” orbital, showing the solidity of the substance and illustrating the chemical activity of the molecule (Figure 6).

Figure 6 has shown the sequence of band energy gap for chemical compounds of natural drugs as ∆E_achillin_ > ∆E_alkannin_ > ∆E_cuminaldehyde_ > ∆E_dillapiole_ > ∆E_estragole_ > ∆E_fenchone_ with the relation coefficient of R^2^ = 0.9374 (Figure 6). While the band energy gap (∆E) decreases, the stability of the compound increases. Therefore, the fenchone is predicted to be more sensitive than other ingredients.

In this research, the energy gap appoints how achillin, alkannin, cuminaldehyde, dillapiole, estragole, and fenchone as an efficient anti-COVID-19 pandemic receptor can be achieved from natural medications. In addition, frontier molecular orbitals carry out an essential function in the optical and electrical properties, such as in UV-Vis spectra [72].

Furthermore, to accomplish more decisive approval in recognizing the compound specifications, a group of chemical reactivity factors containing “µ” (chemical potential), “χ” (electronegativity), “η” (hardness), “ζ” (softness), “ψ” (electrophilicity index) has been accomplished (Table 8) [73,74,75]:

The negative values of “µ” and the positive amounts of other quantities have exhibited good stability of achillin, alkannin, cuminaldehyde, dillapiole, estragole, and fenchone in *Achillea millefolium* (*Yarrow*), *Alkanet*, *Rumex patientia* (*Patience dock*), *Dill*, *Tarragon*, and *Sweet fennel* (Table 8).

### 3.5. Analysis of UV-VIS Spectroscopy

There is a critical factor as an energy gap between “HOMO” and “LUMO” for recognizing the characteristics of molecular electrical transport [76]. Based on the Frank–Condon principle, the maximum absorption peak (max) is related to a UV–visible spectrum to vertical excitation.

**Figure 7:**
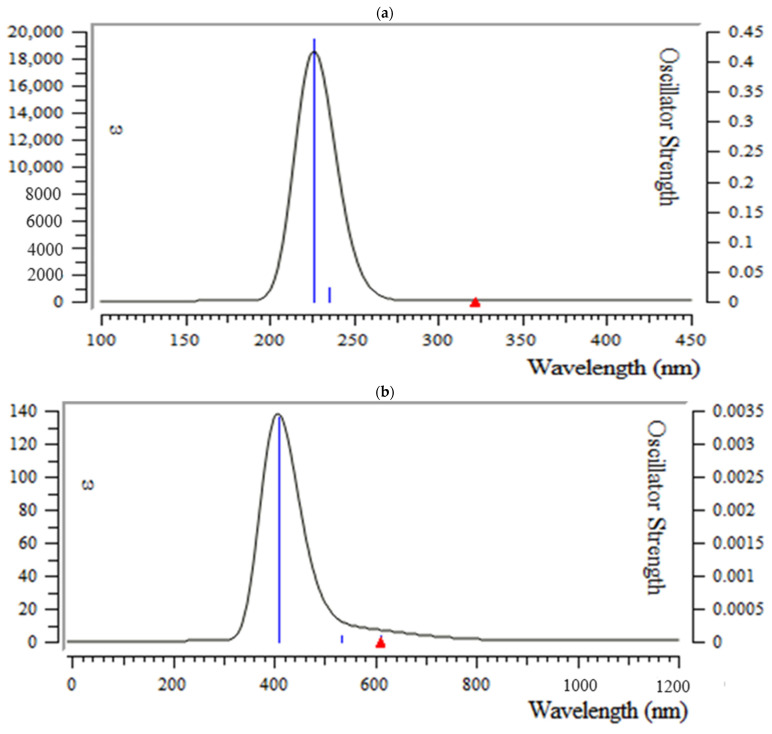

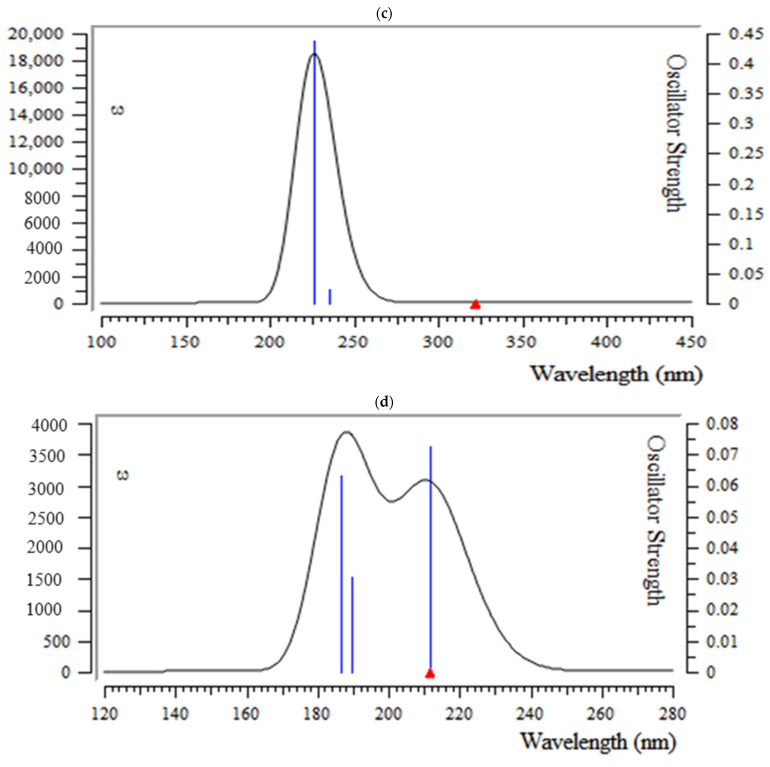

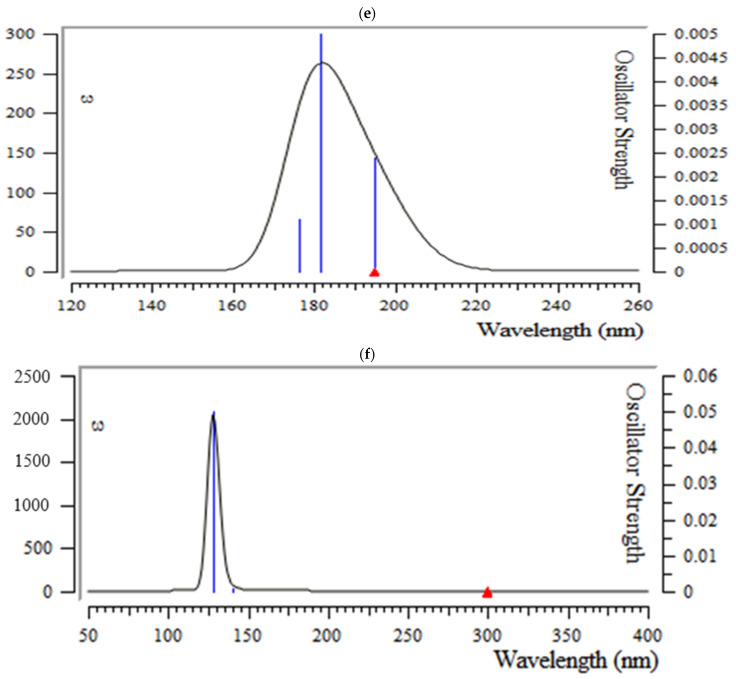
UV–visible spectra of (**a**) achillin, (**b**) alkannin, (**c**) cuminaldehyde, (**d**) dillapiole, (**e**) estragole and (**f**) fenchone.

In this research, TD-DFT/6-311+G (2d,p) computations have been carried out to identify the low-lying excited states of achillin, alkannin, cuminaldehyde, dillapiole, estragole, and fenchone. The results contain the vertical excitation energies, oscillator strength, and wavelength, which have been presented in Figure 7.

In the calculated value of UV–visible spectrum of principal ingredients of medicinal plants, there is a maximum absorption band between 200 and 250 nm for achillin, 250 and 500 nm for alkannin, cuminaldehyde, 170 and 230 nm for dillapiole, 160 and 250 nm for estragole and 100 and 150 nm for Fenchone, respectively. Strong absorption has been observed for achillin, about 225 nm; for alkannin, 488 nm; for cuminaldehyde, 230 nm; and for pamidronic acid, 222 nm, respectively (Figure 7).

This research article has manifested that medicinal plants and phytocompounds can have a considerable function due to their substantial antiviral activity against SARS-CoV-2 and other coronaviruses. Achillin, alkannin, cuminaldehyde, dillapiole, estragole, and Fenchone extracted from *Achillea millefolium* (Yarrow), *Alkanet*, *Rumex patientia* (*Patience dock*), Dill, *Tarragon*, and *Sweet fennel*, respectively, were identified through in silico molecular modeling by using DFT screening. Identified natural phytocompounds revealed to have the potential to exhibit antiviral activities by disrupting the viral life cycle, including viral entrance, replication, assembly, and discharge, as well as virus-specific host targets. Thus, this prompt increase in the pharmaceutical industry focused on phytochemical extracts from medicinal plants and aromatic herbs in the hopes of discovering lead compounds with purposeful antiviral medications.

Here, we used network pharmacology, metabolite analysis, and molecular simulation to comprehend the biochemical basis of the health-boosting impact of medicinal plants. The present study investigates the drug ability, metabolites, and potential interaction of the title tea with genes associated with COVID-19-induced pathogenesis.

## 4. Conclusions

Medicinal plants of achillin, alkannin, cuminaldehyde, dillapiole, estragole, and fenchone are puissant to adhere the database amino acids segment of Tyr160-Met161-His162 agent as the appointive area of the COVID-19 through exhibiting the alteration in their frequency and intensity spectra after approximation by “NMR” approach which is influenced by the atomic configuration of the anti-virus macromolecule. The resistance of hydrogen bonding between several pharmaceutical extracts of achillin, alkannin, cuminaldehyde, dillapiole, estragole, fenchone, and COVID-19 through the constitution of anti-COVID-19 through two possibilities of [N
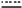
H] and [O
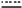
H] with distinct atomic charges have been inquired using “IR” approaches. Therefore, the thermodynamic attributes of Gibbs free energy, enthalpy of formation, Electronic Energy, and Core–Core interaction can authorize the consistency of anti-COVID-19 due to hydrogen bonding foundation using the drug design framework. Moreover, the lowering of the energy gap [∆E = E_LUMO_ –E_HOMO_] has illustrated the charge transfer interactions taking place within achillin, alkannin, cuminaldehyde, dillapiole, estragole, and fenchone. The atomic charges have donated the proper perception of molecular theory and the energies of fundamental molecular orbitals.

## Figures and Tables

**Table 1:** Plant species most preferred against COVID-19 include achillin, alkannin, cuminaldehyde, dillapiole, estragole, and fenchone.

Compound	Molecular Structure	Sources	Applied Symptom
Achillin	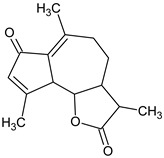	*Achillea millefolium* (*Yarrow*)	weakness, cough, sore throat, nausea-vomiting
Alkannin	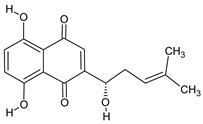	*Alkanet*	skin rash, diarrhea
Cuminaldehyde	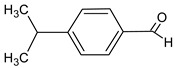	*Rumex patientia* (*Patience dock*)	skin rash, sore throat, fever
*Dill*apiole	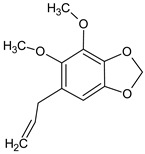	*Dill*	anorexia
Estragole	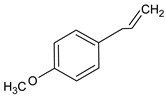	*Tarragon*	fever, muscle-joint pain, anorexia
Fenchone	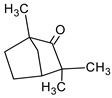	*Sweet fennel*	shortness of breath

**Table 2:** The geometry optimization amounts with m062x/cc-pvdz pseudo=CEP for achillin, cuminaldehyde, dillapiole, and estragole bound to the active site of COVID-19 protein through the drug design approach.

Medicinal Extracts—COVID-19 Active Area	Bond Length	(Å)	Bond/Torsion Angle	(°)
Achillin	N67-H68	1.036	N67-H68-O15	176.181
	H68-O15	0.9974		
	O15-C13	1.4123	N67-H68-O15-C13	178.492
Cuminaldehyde	N61-H62	1.0351	N61-H62-O9	179.192
	H62-O9	0.9966		
	O9-C7	1.4150	N61-H62-O9-C7	31.2731
Dillapiole	N78-H79	1.0295	N78-H79-C13	179.216
	H79-C13	1.1193		
	C13-O12	1.4131	N78-H79-C13-O12	55.0114
Estragole	N71-H72	1.0358	N71-H72-C11	179.208
	H72-C11	1.1244		
	C11-O10	1.4099	N71-H72-C11-O10	106.924

**Table 3:** The compounds of Achillin, cuminaldehyde, dillapiole, and estragole as anti-COVID-19 drugs in distinct normal modes of infrared spectra.

Inhibitor	Normal Mode	Frequency (1/cm)	Intensity (km/mol)
Achillin	275	3336.01	2292.987
Cuminaldehyde	236	3395.38	2270.866
Dillapiole	205	1998.66	202.722
Estragole	185	1971.26	226.961

**Table 4:** The thermodynamic factors of achillin, alkannin, cuminaldehyde, dillapiole, and estragole bound to COVID-19 active site protein.

Plant Component—Active Site	∆G × 10^−4^ (kcal/mol)	∆S (kcal/K.mol)	E_electronic_ × 10^−4^ (kcal/mol)	E_core-core_ × 10^−4^ (kcal/mol)
Achillin	−18.2174	607.2787	−214.3615	196.1441
Alkannin	−19.6971	656.5647	−232.3721	212.6750
Cuminaldehyde	−15.2850	509.7272	−165.8901	150.6051
Dillapiole	−17.8553	595.2878	−198.4780	180.6227
Estragole	−15.2109	507.3701	−160.6792	145.4682

**Table 5:** The calculated difference in ∆H_F_ (kcal/mol) (heat formation) for achillin, alkannin, cuminaldehyde, dillapiole, and estragole attached to the critical point of “TMH” derived from COVID-19 macromolecule at 300 K.

**∆H_TMH_ × 10^−4^** 25.8242 (kcal/mol)	**∆H _Achillin_**	**∆H_(Achillin - active site_**_)_	**∆H_F_ × 10^−4^ = ∆H _(Achillin - active site__)_ – (∆H _Achillin_ + ∆H _active site_)**
	−76.2424	9.5452	−25.8156
	**∆H _Alkannin_**	**∆H_(Alkannin - active site_**_)_	**∆H_F_ × 10^−4^ = ∆H _(Alkannin - active site__)_ – (∆H _Alkannin_ + ∆H _active site_)**
	−80.8417	−1.3898	−25.8162
	**∆H _Cuminaldehyde_**	**∆H_(Cuminaldehyde - active site_**_)_	**∆H_F_ × 10^−4^ = ∆H _(Cuminaldehyde - active site__)_ – (∆H _Cuminaldehyde_ +∆H _active site_)**
	−3.6680	67.8448	−25.8170
	**∆H _Dillapiole_**	**∆H_(Dillapiole - active site_**_)_	**∆H_F_ × 10^−4^ = ∆H_(Dillapiole - active site__)_ – (∆H _Dillapiole_ + ∆H _active site_)**
	−31.3428	33.0993	−25.8177
	**∆H _Estragole_**	**∆H _(Estragole - active site_**_)_	**∆H_F_ × 10^−4^ =∆H _(Estragole - active site__)_ – (∆H _Estragole_ + ∆H_active site_)**
	101.5614	14.9017	−25.8328

**Table 6:** The amounts of atomic charge (Q) for indicated “O” atoms in the linkage of achillin, alkannin, cuminaldehyde, dillapiole, and estragole to Tyr160-Met161-His162.

Achillin	Q	Alkannin	Q	Cuminaldehyde	Q	Dillapiole	Q	Estragole	Q
N19	−0.0463	N24	−0.0442	N13	−0.0450	N30	−0.0455	N23	−0.0463
N40	−0.0506	N45	−0.0513	N34	−0.0503	N51	−0.0501	N44	−0.0482
N57	−0.0323	N62	−0.0368	N51	−0.0375	N68	−0.0377	N61	−0.0367
N67	0.1071	N72	0.0057	N61	0.0505	N78	0.3846	N71	0.2459
N73	−0.1103	N78	−0.0961	N67	−0.1211	N84	−0.0413	N77	−0.0487
O14	−0.2489	O11	−0.4572	O9	−0.4548	O9	−0.1526	O10	−0.1878
O15	−0.4204	O12	−0.1939	O18	−0.4041	O10	−0.1758	O28	−0.4031
O24	−0.4027	O14	−0.3115	O32	−0.2455	O12	−0.1684	O42	−0.2305
O38	−0.2326	O21	−0.3452	O39	−0.3786	O49	−0.2174	O49	−0.3875
O45	−0.3872	O29	−0.4001	O56	−0.3090	O56	−0.3826	O66	−0.3223
O62	−0.3070	O43	−0.2657			O73	−0.3254		
		O50	−0.3842						
		O67	−0.2939						

**Table 7:** The “HOMO” (a.u.), “LUMO” (a.u.), and band energy gap (eV) of achillin, alkannin, cuminaldehyde, dillapiole, estragole, and fenchone.

Molecule	E_LUMO_ (a.u.)		E_HOMO_ (a.u.)		∆E = E_LUMO_ – E_HOMO_ (eV)
Achilin	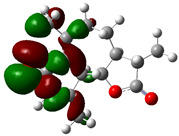	−0.0266	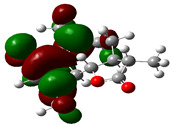	−0.3019	7.4899
Alkannin	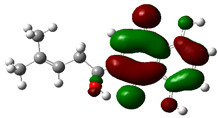	−0.0283	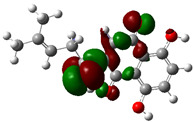	−0.1767	4.0381
Cuminaldehyde	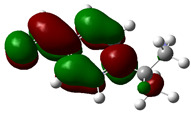	−0.0887	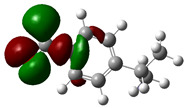	−0.2075	3.2327
Dillapiole	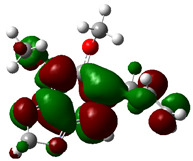	−0.1575	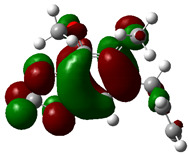	−0.1717	0.3864
Estragole	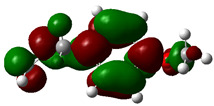	−0.1523	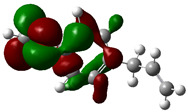	−0.2141	1.6816
Fenchone	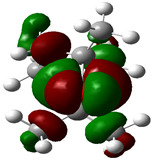	−0.1605	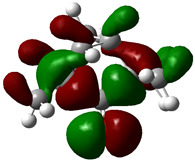	−0.1907	0.8218

**Table 8:** Chemical reactivity factors “µ”, “χ”, “η”, “ζ”, “ψ” have been accomplished using the following equations for achillin, alkannin, cuminaldehyde, dillapiole, estragole, and fenchone.

Compounds	µ = (E_HOMO_ + E_LUMO_)/2	χ = –(E_HOMO_ + E_LUMO_)/2	η = (E_LUMO_–E_HOMO_)/2	ζ = 1/(2η)	ψ = µ^2^/(2η)
Achilin	−4.4694	4.4694	3.74495	0.1335	2.6670
alkannin	−2.7891	2.7891	2.01905	0.2476	1.9264
cuminaldehyde	−4.0300	4.0300	1.61635	0.3093	5.0239
dillapiole	−4.4790	4.4790	0.1932	2.5880	51.9188
estragole	−4.9851	4.9851	0.8408	0.5947	14.7783
fenchone	−4.7783	4.7783	0.8408	0.5947	13.5776

## Data Availability

Data are contained within this article.

## References

[1] Alomair L., Mustafa S., Jafri M.S., Alharbi W., Aljouie A., Almsned F., Alawad M., Bokhari Y.A., Rashid M. (2022). Molecular Dynamics Simulations to Decipher the Role of Phosphorylation of SARS-CoV-2 Nonstructural Proteins (nsps) in Viral Replication. Viruses.

[2] Plavec Z., Domanska A., Liu X., Laine P., Paulin L., Varjosalo M., Auvinen P., Wolf S.G., Anastasina M., Butcher S.J. (2022). SARS-CoV-2 Production, Purification Methods and UV Inactivation for Proteomics and Structural Studies. Viruses.

[3] Monajjemi M., Shahriari S., Mollaamin F. (2020). Evaluation of Coronavirus Families & COVID-19 Proteins: Molecular Modeling Study. Biointerface Res. Appl. Chem..

[4] Yarovaya O.I., Shcherbakov D.N., Borisevich S.S., Sokolova A.S., Gureev M.A., Khamitov E.M., Rudometova N.B., Zybkina A.V., Mordvinova E.D., Zaykovskaya A.V. (2022). Borneol Ester Derivatives as Entry Inhibitors of a Wide Spectrum of SARS-CoV-2 Viruses. Viruses.

[5] Shahriari S., Monajjemi M., Mollaamin F. (2022). Determination of proteins specification with SARS-COVID-19 based ligand designing. J. Chil. Chem. Soc..

[6] Majeed A., Zhang X. (2023). On the Adoption of Modern Technologies to Fight the COVID-19 Pandemic: A Technical Synthesis of Latest Developments. COVID.

[7] Bonaccorsi G., Pierri F., Cinelli M., Flori A., Galeazzi A., Porcelli F., Schmidt A.L., Valensise C.M., Scala A., Quattrociocchi W. (2020). Economic and social consequences of human mobility restrictions under COVID-19. Proc. Natl. Acad. Sci. USA.

[8] Barakat A., Mostafa A., Ali M., Al-Majid A.M., Domingo L.R., Kutkat O., Moatasim Y., Zia K., Ul-Haq Z., Elshaier Y.A.M.M. (2022). Design, Synthesis and In Vitro Evaluation of Spirooxindole-Based Phenylsulfonyl Moiety as a Candidate Anti-SAR-CoV-2 and MERS-CoV-2 with the Implementation of Combination Studies. Int. J. Mol. Sci..

[9] Mollaamin F., Monajjemi M. (2021). Thermodynamic research on the inhibitors of coronavirus through drug delivery method. J. Chil. Chem. Soc..

[10] Sardar T., Nadim S.S., Rana S., Chattopadhyay J. (2020). Assessment of lockdown effect in some states and overall India: A predictive mathematical study on COVID-19 outbreak. Chaos Solitons Fract..

[11] Mollaamin F., Shahriari S., Monajjemi M. (2023). Treating omicron BA.4 & BA.5 via herbal antioxidant asafoetida: A DFT study of carbon nanocarrier in drug delivery. J. Chil. Chem. Soc..

[12] Zeng F., Huang Y., Guo Y., Yin M., Chen X., Xiao L., Deng G. (2020). Association of inflammatory markers with the severity of COVID-19: A meta-analysis. Int. J. Infect. Dis..

[13] Mollaamin F. (2022). Physicochemical investigation of anti-COVID19 drugs using several medicinal plants. J. Chil. Chem. Soc..

[14] Jamal Q.M.S. (2022). Antiviral Potential of Plants against COVID-19 during Outbreaks—An Update. Int. J. Mol. Sci..

[15] Remali J., Aizat W.M. (2021). A review on plant bioactive compounds and their modes of action against coronavirus infection. Front. Pharmacol..

[16] Singh S., Sk M.F., Sonawane A., Kar P., Sadhukhan S. (2021). Plant-derived natural polyphenols as potential antiviral drugs against SARS-CoV-2 via rna-dependent RNA polymerase (rdrp) inhibition: An in-silico analysis. J. Biomol. Struct. Dyn..

[17] Capell T., Twyman R.M., Armario-Najera V., Ma J.K.-C., Schillberg S., Christou P. (2020). Potential applications of plant biotechnology against SARS-CoV-2. Trends Plant Sci..

[18] Mollaamin F. (2021). Function of anti-cov structure using inh [1-6]-tyr160-met161-his162 complex. Biointerface Res. Appl. Chem..

[19] Bibi S., Khan M.S., El-Kafrawy S.A., Alandijany T.A., El-Daly M.M., Yousafi Q., Fatima D., Faizo A.A., Bajrai L.H., Azhar E.I. (2022). Virtual screening and molecular dynamics simulation analysis of Forsythoside A as a plant-derived inhibitor of SARS-CoV-2 3clpro. Saudi Pharm. J..

[20] Raudone L., Vilkickyte G., Marksa M., Radusiene J. (2024). Comparative Phytoprofiling of Achillea millefolium Morphotypes: Assessing Antioxidant Activity, Phenolic and Triterpenic Compounds Variation across Different Plant Parts. Plants.

[21] Faran M., Tcherni A. (1997). Medicinal Herbs in Modern Medicine (Ṣimḥei Marpé Bir’fū’ah ha-Modernīt).

[22] Martin C. (2012). How to Grow Perennial Vegetables.

[23] Rojas-Martínez R., Arrieta J., Cruz-Antonio L., Arrieta-Baez D., Velázquez-Méndez A.M., Sánchez-Mendoza M.E. (2013). Dillapiole, Isolated from Peperomia pellucida, Shows Gastroprotector Activity against Ethanol-Induced Gastric Lesions in Wistar Rats. Molecules..

[24] Schepetkin I.A., Özek G., Özek T., Kirpotina L.N., Klein R.A., Khlebnikov A.I., Quinn M.T. (2023). Composition and Biological Activity of the Essential Oils fromWild Horsemint, Yarrow, and Yampah from Subalpine Meadows in Southwestern Montana: Immunomodulatory Activity of Dillapiole. Plants.

[25] Shultz L.M., Flora of North America Editorial Committee (2006). *Artemisia dracunculus*.. Flora of North America North of Mexico (FNA).

[26] Zeller A., Rychlik M. (2007). Impact of estragole and other odorants on the flavour of anise and tarragon. Flavour Fragr. J..

[27] Ribeiro-Santos R., Andrade M., Sanches-Silva A., de Melo N.R. (2017). Essential Oils for Food Application: Natural Substances with Established Biological Activities. Food Bioprocess Technol..

[28] Badgujar S.B., Patel V.V., Bandivdekar A.H. (2014). *Foeniculum vulgare* Mill: A Review of Its Botany, Phytochemistry, Pharmacology, Contemporary Application, and Toxicology. BioMed Res. Int..

[29] Zakaryan H., Arabyan E., Oo A., Zandi K. (2017). Flavonoids: Promising natural compounds against viral infections. Arch. Virol..

[30] Seema T.M., Thyagarajan S.P. (2016). Pa-9: A flavonoid extracted from plectranthus amboinicus inhibits HIV-1 protease. Int. J. Pharmacogn. Phytochem. Res..

[31] Jo S., Kim S., Shin D.H., Kim M.S. (2020). Inhibition of SARS-CoV 3CL protease by flavonoids. J. Enzyme Inhib. Med. Chem..

[32] Dhama K., Natesan S., Iqbal Yatoo M., Patel S.K., Tiwari R., Saxena S.K., Harapan H. (2020). Plant-based vaccines and antibodies to combat COVID-19: Current status and prospects. Hum. Vaccines Immunother..

[33] Nawrot-Hadzik I., Zmudzinski M., Matkowski A., Preissner R., Kęsik-Brodacka M., Hadzik J., Drag M., Abel R. (2021). *Reynoutria* Rhizomes as a Natural Source of SARS-CoV-2 Mpro Inhibitors–Molecular Docking and In Vitro Study. Pharmaceuticals.

[34] Dwarka D., Agoni C., Mellem J.J., Soliman M.E., Baijnath H. (2020). Identification of potential SARS-CoV-2 inhibitors from South African medicinal plant extracts using molecular modelling approaches. S. Afr. J. Bot..

[35] Kulkarni S.A., Nagarajan S.K., Ramesh V., Palaniyandi V., Selvam S.P., Madhavan T. (2020). Computational evaluation of major components from plant essential oils as potent inhibitors of SARS-CoV-2 spike protein. J. Mol. Struct..

[36] Shree P., Mishra P., Selvaraj C., Singh S.K., Chaube R., Garg N., Tripathi Y.B. (2020). Targeting COVID-19 (SARS-CoV-2) main protease through active phytochemicals of ayurvedic medicinal plants—*Withania somnifera* (Ashwagandha), *Tinospora cordifolia* (Giloy) and *Ocimum sanctum* (Tulsi)—A molecular docking study. J. Biomol. Struct. Dyn..

[37] Nawrot J., Gornowicz-Porowska J., Budzianowski J., Nowak G., Schroeder G., Kurczewska J. (2022). Medicinal Herbs in the Relief of Neurological, Cardiovascular, and Respiratory Symptoms after COVID-19 Infection A Literature Review. Cells.

[38] Frisch M.J., Trucks G.W., Schlegel H.B., Scuseria G.E., Robb M.A., Cheeseman J.R., Scalmani G., Barone V., Petersson G.A., Nakatsuji H. (2016). Gaussian 16, Revision C.01.

[39] Perdew J.P., Burke K., Ernzerhof M. (1996). Generalized Gradient Approximation Made Simple. Phys. Rev. Lett..

[40] Bakhshi K., Mollaamin F., Monajjemi M. (2011). Exchange and correlation effect of hydrogen chemisorption on nano V(100) surface: A DFT study by generalized gradient approximation (GGA). J. Comput. Theor. Nanosci..

[41] Monajjemi M., Mahdavian L., Mollaamin F., Khaleghian M. (2009). Interaction of Na, Mg, Al, Si with carbon nanotube (CNT): NMR and IR study. Russ. J. Inorg. Chem..

[42] Mollaamin F., Shahriari S., Monajjemi M. (2022). Drug design of medicinal plants as a treatment of omicron variant (COVID-19 variant B.1.1.529). J. Chil. Chem. Soc..

[43] Monajjemi M., Noei M., Mollaamin F. (2010). Design of fMet-tRNA and Calculation of its Bonding Properties by Quantum Mechanics. Nucleosides Nucleotides Nucleic Acids.

[44] Mollaamin F., Monajjemi M. (2015). Harmonic Linear Combination and Normal Mode Analysis of Semiconductor Nanotubes Vibrations. J. Comput. Theor. Nanosci.

[45] Khaleghian M., Zahmatkesh M., Mollaamin F., Monajjemi M. (2011). Investigation of Solvent Effects on Armchair Single-Walled Carbon Nanotubes: A QM/MD Study. Fuller. Nanotub. Carbon Nanostruct..

[46] Sarasia E.M., Afsharnezhad S., Honarparvar B., Mollaamin F., Monajjemi M. (2011). Theoretical study of solvent effect on NMR shielding tensors of luciferin derivatives. Phys. Chem. Liquids.

[47] Mollaamin F., Monajjemi M. (2024). In Situ Ti-Embedded SiC as Chemiresistive Nanosensor for Safety Monitoring of CO, CO_2_, NO, NO_2_: Molecular Modelling by Conceptual Density Functional Theory. Russ. J. Phys. Chem. B.

[48] Tahan A., Mollaamin F., Monajjemi M. (2009). Thermochemistry and NBO analysis of peptide bond: Investigation of basis sets and binding energy. Russ. J. Phys. Chem. A.

[49] Mollaamin F., Shahriari S., Monajjemi M. (2023). Monkeypox disease treatment by tecovirimat adsorbed onto single-walled carbon nanotube through drug delivery method. J. Chil. Chem. Soc..

[50] Mollaamin F., Monajjemi M. (2023). Molecular modelling framework of metal-organic clusters for conserving surfaces: Langmuir sorption through the TD-DFT/ONIOM approach. Mol. Simul..

[51] Shahriari S., Mollaamin F., Monajjemi M. (2023). Increasing the Performance of {[(1-x-y) LiCo_0.3_Cu_0.7_] (Al and Mg doped)] O_2_}, xLi_2_MnO_3_, yLiCoO_2_ Composites as Cathode Material in Lithium-Ion Battery: Synthesis and Characterization. Micromachines.

[52] McArdle S., Mayorov A., Shan X., Benjamin S., Yuan X. (2019). Digital quantum simulation of molecular vibrations. Chem. Sci..

[53] Monajjemi M., Baie M.T., Mollaamin F. (2010). Interaction between threonine and cadmium cation in [Cd(Thr)] (n = 1–3) complexes: Density functional calculations. Russ Chem Bull..

[54] Zadeh M.A.A., Lari H., Kharghanian L., Balali E., Khadivi R., Yahyaei H., Mollaamin F., Monajjemi M. (2015). Density functional theory study and anti-cancer properties of shyshaq plant: In view point of nano biotechnology. J. Comput. Theor. Nanosci..

[55] Ciliberto G., Cardone L. (2020). Boosting the arsenal against COVID-19 through computational drug repurposing. Drug Discov. Today.

[56] Hatada R., Okuwaki K., Mochizuki Y., Handa Y., Fukuzawa K., Komeiji Y., Okiyama Y., Tanaka S. (2020). Fragment molecular orbital based interaction analyses on COVID-19 main protease-inhibitor N3 complex (PDB ID: 6LU7). J. Chem. Inf. Model..

[57] Peele K.A., Chandrasai P., Srihansa T., Krupanidhi S., Sai A.V., Babu D.J., Indira M., Reddy A.R., Venkateswarulu T. (2020). Molecular docking and dynamic simulations for antiviral compounds against SARS-CoV-2: A computational study. Inform. Med. Unlocked.

[58] Qiao Z., Zhang H., Ji H.-F., Chen Q. (2020). Computational view toward the inhibition of SARS-CoV-2 spike glycoprotein and the 3CL protease. Computation.

[59] Liang J., Pitsillou E., Karagiannis C., Darmawan K.K., Ng K., Hung A., Karagiannis T.C. (2020). Interaction of the prototypical α-ketoamide inhibitor with the SARS-CoV-2 main protease active site in silico: Molecular dynamic simulations highlight the stability of the ligand-protein complex. Comput. Biol. Chem..

[60] Mollaamin F. (2023). Conocimiento de enfermedades virales terapéuticas: Aplicación de SWCNT en la administración de fármacos. Rev. Colomb. Quim..

[61] Monajjemi M., Mollaamin F., Shojaei S. (2020). An overview on coronaviruses family from past to COVID-19: Introduce some inhibitors as antiviruses from Gillan’s plants. Biointerface Res. Appl. Chem..

[62] Mollaamin F., Monajjemi M. (2024). Adsorption ability of Ga5N10 nanomaterial for removing metal ions contamination from drinking water by DFT. Int. J. Quantum Chem..

[63] Wang S. (2019). Efficiently Calculating Anharmonic Frequencies of Molecular Vibration by Molecular Dynamics Trajectory Analysis. ACS Omega.

[64] Mollaamin F., Monajjemi M., Salemi S., Baei M.T. (2011). A Dielectric Effect on Normal Mode Analysis and Symmetry of BNNT Nanotube. Fuller. Nanotub. Carbon Nanostruct..

[65] Mollaamin F., Monajjemi M. (2024). Trapping of toxic heavy metals from water by GN–nanocage: Application of nanomaterials for contaminant removal technique. J. Mol. Struct..

[66] Mollaamin F., Monajjemi M. (2023). Transition metal (X = Mn, Fe, Co, Ni, Cu, Zn)-doped graphene as gas sensor for CO_2_ and NO_2_ detection: A molecular modeling framework by DFT perspective. J. Mol. Model..

[67] Aji G.K., Hatou K., Morimoto T. (2020). Modeling the Dynamic Response of Plant Growth to Root Zone Temperature in Hydroponic Chili Pepper Plant Using Neural Networks. Agriculture.

[68] Mollaamin F., Monajjemi M. (2023). Tailoring and functionalizing the graphitic-like GaN and GaP nanostructures as selective sensors for NO, NO_2_, and NH_3_ adsorbing: A DFT study. J. Mol. Model..

[69] Khalili Hadad B., Mollaamin F., Monajjemi M. (2022). Biophysical chemistry of macrocycles for drug delivery: A theoretical study. Russ. Chem. Bull..

[70] Mollaamin F., Ilkhani A., Sakhaei N., Bonsakhteh B., Faridchehr A., Tohidi S., Monajjemi M. (2015). Thermodynamic and solvent effect on dynamic structures of nano bilayer-cell membrane: Hydrogen bonding study. J. Comput. Theor. Nanosci..

[71] Monajjemi M., Khaleghian M., Tadayonpour N., Mollaamin F. (2010). The effect of different solvents and temperatures on stability of single-walled carbon nanotube: A QM/MD study. Int. J. Nanosci..

[72] Aihara J. (1999). Reduced HOMO−LUMO Gap as an Index of Kinetic Stability for Polycyclic Aromatic Hydrocarbons. J. Phys. Chem. A.

[73] Mollaamin F., Monajjemi M. (2023). In Silico-DFT Investigation of Nanocluster Alloys of Al-(Mg,Ge,Sn) Coated by Nitrogen Heterocyclic Carbenes as Corrosion Inhibitors. J. Clust. Sci..

[74] Parr R.G., Pearson R.G. (1983). Absolute Hardness: Companion Parameter to Absolute Electronegativity. J. Am. Chem. Soc..

[75] Politzer P., Abu-Awwad F. (1998). A comparative analysis of Hartree-Fock and Kohn-Sham orbital energies. Theor. Chem. Acc..

[76] Mollaamin F., Shahriari S., Monajjemi M., Khalaj Z. (2023). Nanocluster of Aluminum Lattice via Organic Inhibitors Coating: A Study of Freundlich Adsorption. J. Clust. Sci..

